# Does the structure of light influence the speckle size?

**DOI:** 10.1038/s41598-019-56964-0

**Published:** 2020-01-13

**Authors:** Xiao-Bo Hu, Meng-Xuan Dong, Zhi-Han Zhu, Wei Gao, Carmelo Rosales-Guzmán

**Affiliations:** 1 The Higher Educational Key Laboratory for Measuring & Control Technology and Instrumentations of Heilongjiang Province, Harbin University of Science & Technology, Harbin, 150080 China; 2 Wang Da-Heng Collaborative Innovation Center, Heilongjiang Provincial Key Laboratory of Quantum manipulation & Control, Harbin University of Science and Technology, Harbin, 150080 China

**Keywords:** Optical metrology, Liquid crystals

## Abstract

It is well known that when a laser is reflected from a rough surface or transmitted through a diffusive medium, a speckle pattern will be formed at a given observation plane. An important parameter of speckle is its size, which for the case of homogeneous illumination, well-known relations for its computation have been derived. This is not the case for structured light beams of non-homogeneous intensity and phase distribution. Here, we propose and demonstrate, using Hermite- and Laguerre-Gaussian light modes, that the mean size of the speckle generated by these structured light beams can be measured assuming a homogeneous illumination. We further provide with mathematical expressions that relate the speckle size to the generalised definition of "spot size". To reinforce our assessment, we compare the mean speckle size generated by structured light modes with that generated by wave fronts of constant phase and amplitude and show that in both cases the mean speckle size is almost identical. Our findings reveal a fundamental property of speckle, which will be of great relevance in many speckle-based applications and will pave the way towards the development of novel applications.

## Introduction

As almost as early as the invention of the laser, an unexpected phenomenon produced when a rough surface is illuminated by a coherence light source became ubiquitous, the formation of a grainy structure of bright spots of light and darkness known as speckle, a phenomenon already known since the time of Isaac Newton. Speckle can be explained using the Huygens principle by considering the microscopic structure of the surface^1,2^. In essence, the scattered optical wave, at a later distance after the rough surface, may be considered as the superposition of a large number of coherent wavelets with random phases emerging from the various microscopic elements on the surface that generates regions of constructive and destructive interference. Importantly, although in its origins speckle was regarded as an undesired noise that should be removed, in particular for digital holography^3^, it rapidly became a powerful optical tool in a myriad of applications^4–11^. Moreover, in recent time, there has been an increasing interest in developing novel techniques for the generation of customised speckle, either with a tailored speckle-size distribution^12–16^, with exotic properties, such as, non-diffraction or self-healing^17–20^, or even with vectorial properties^21^. Crucially, a proper characterisation and control of speckle is highly desirable, not only in the development of applications but also in the generation of customised speckle. Along this line, an important parameter is its size, which for simple cases, such as illumination by circular or rectangular apertures of homogeneous intensity, well-known mathematical expressions have been derived for its computation. This is not the case of speckle produced by structured light beams of non-homogeneous intensity and phase distribution. Pioneering studies along this direction have restricted to the specific case of optical vortices characterised by a ring-like intensity distribution and a helical phase of the form $\exp i\ell \phi$, where $\ell \in {\mathbb{Z}}$ is known as the topological charge^22^. These studies reported that a decrease of the speckle size was due to an increase of the topological charge, which caused an increase in the size of the optical vortex. Further, the authors proposed a complicated method to measure the ring-like area of the optical vortex to provide with a mathematical expression relating this to the speckle size. Additional studies considered optical vortices of varying topological charge but constant size, so-called perfect optical vortices^23–25^, to demonstrate that the speckle size did not depend on the topological charge^20^.

Here, we use a more general class of structured light beams to study the statistical properties of the speckle size. More precisely, we use well-known solutions of the paraxial wave equation, the Laguerre-Gaussian ($L{G}_{p}^{\ell }$) and Hermite-Gaussian ($H{G}_{n}^{m}$) modes of light to show that their inner structure does not influence the speckle size. The first set of modes, obtained in the cylindrical coordinates ($\rho ,\varphi$), is characterised by a complex intensity distribution of $p+1(p\in {\mathbb{N}})$ concentric rings of varying intensity, and a non-constant phase. The second, obtained in the Cartesian coordinates (x,y), features a rectangular structure of $(m+1)\times (n+1)$ vertical and horizontal spots of light, where, $n$ and $m\in {\mathbb{N}}$. Our studies reveal that the size of the generated speckle is uniquely related to the total illuminated area, rather than to the complex structure embedded in their phase and amplitude distribution. We further corroborated our findings by comparing the speckle size of the structured beams with rectangular and circular apertures of homogeneous intensity and constant phase finding a perfect match between our theoretical model and both types of illuminations, structured and homogeneous.

## Theoretical Background

### Statistical measure of the speckle size

To start with, it is worth mentioning that speckles do not have a well-defined size, hence, we can only provide with a measure of the mean speckle size. Further, even though the statistical properties of speckle depend on the coherence of the incident light and the detailed properties of the random surface or medium, for perfectly coherent light and for a surface whose roughness is in the order or greater than the wavelength of the illumination source, this dependence on the random scatterer is almost negligible. Under this condition, a measure of the mean speckle size can be done through the normalised autocorrelation function of the speckle intensity at a given observation plane, namely^26^,

1 $$C(\Delta u,\Delta v)=\langle I({u}_{1},{v}_{1})I({u}_{2},{v}_{2})\rangle ,$$

where, $\Delta u={u}_{2}-{u}_{1}$, and $\Delta v={v}_{2}-{v}_{1}$. Here, $I({u}_{i},{v}_{i})$, $i=1,2$, is the intensity at two points in the observation plane resulting from the interference of a large number of emitters in the scattering surface that absorbs and re-emits the incident light wave. Additionally, $\langle \rangle$ represents the spatial average over a large number of speckles in the observation plane. The mean speckle size is then defined as the distance $\Delta s$ at which both intensities become uncorrelated to one another. A mathematical expression for C $(\Delta u,\Delta v)$ in terms of the intensity distribution $|P(x,y){|}^{2}$ of the field impinging on the scattering surface is given by^26^,

2 $$C\,(\Delta u,\Delta v)={\langle I\rangle }^{2}\,[1+{|\frac{{\int }_{-}^{+}{\int }_{\infty }^{\infty }|P(x,y){|}^{2}\exp [\frac{i2\pi }{\lambda {z}_{f}}(x\Delta u+y\Delta v)]{\rm{d}}x{\rm{d}}y}{{\int }_{-}^{+}{\int }_{\infty }^{\infty }|P(x,y){|}^{2}{\rm{d}}x{\rm{d}}y}|}^{2}].$$

and is the basis of our study. The integral in the numerator is in essence the Fourier transform of $|P(x,y){|}^{2}$, whereas the integral in the denominator is the total power of the incident field.

**Figure 1:**
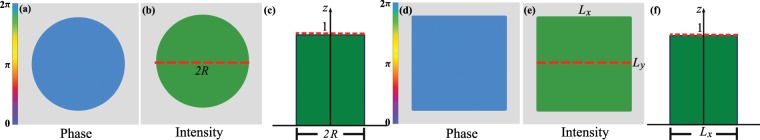
Phase (**a**) and amplitude (**b**) of a plane wave illuminating a circular aperture of radius $R$. (**c**) Intensity distribution along the dashed line. Phase (**d**) and amplitude (**e**) of a plane wave illuminating a rectangular aperture of dimensions ${L}_{x}\times {L}_{y}$. (**f**) Intensity distribution along the dashed line. Notice that in both cases the amplitude and the phase are homogeneous.

This expression can be used to determine the speckle size produced by laser beams with simple structures, such as, Gaussian or plane waves^27,28^. In particular, for the specific case of a unit-amplitude plane wave of constant phase and homogeneous intensity illuminating a circular region of radius *R*, see Fig. 1(a–c), the mean speckle size as function of the illuminated area is given by (see Supplementary Materials),

3 $$\Delta {s}_{circ}=\frac{1.22\sqrt{\pi }\lambda {z}_{f}}{2\sqrt{{A}_{circ}}}$$

where ${z}_{f}$ is the distance from the scattering surface to the observation plane and ${A}_{circ}=\pi {R}^{2}$ is the total area illuminating the surface. A similar expression can be found for a rectangular aperture of area ${A}_{rect}={L}_{x}{L}_{y}$, (see Fig. 1(d–f)), namely (see Supplementary Materials),

4 $$\Delta {s}_{rect}=\frac{\lambda {z}_{f}}{\sqrt{{A}_{rect}}},$$

Importantly, the relations in Eqs. 3 and 4 show that the average speckle size increases linearly with the distance ${z}_{f}$ from the scattering surface to the observation plane and decreases as the illuminated area increases. Notice also that Eqs. 3 and 4 differ by the factor $1.22\sqrt{\pi }/2$, which comes from the difference in the geometry of both apertures.

### The Laguerre- and Hermite-Gaussian modes

**Figure 2:**
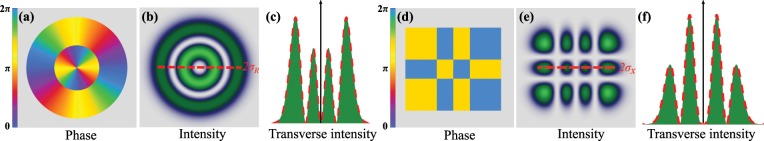
Phase (**a**) and intensity distribution (**b**) of an $L{G}_{1}^{2}$ mode. (**c**) Intensity profile along the dashed line. Phase (**d**) intensity distribution (**e**) of an $H{G}_{23}$ mode. (**f**) Intensity profile along the dashed line. Notice how in both cases the phase (bar on the left) and the intensity distribution feature a non-homogeneous distribution.

The $L{G}_{p}^{\ell }$ modes can be described mathematically in terms of the Laguerre polynomials ${L}_{p}^{\ell }(\rho ,\varphi )$ as,

5 $$\begin{array}{rcl}L{G}_{p}^{\ell }(\rho ,\varphi ,z) & = & \sqrt{\frac{2p!}{\pi (|\ell |+p)!{\omega }^{2}(z)}}{[\frac{\sqrt{2}\rho }{\omega (z)}]}^{|\ell |}{L}_{p}^{|\ell |}\,[\frac{2{\rho }^{2}}{{\omega }^{2}(z)}]\,\exp \,[i(2p+|\ell |+1)\zeta (z)]\\  &  & \exp \,[-\frac{{\rho }^{2}}{{\omega }^{2}(z)}]\,\exp \,[\frac{-ik{\rho }^{2}}{2R(z)}]\,\exp \,[\,-\,i\ell \varphi ]\,\exp \,[\,-\,ikz].\end{array}$$

where $\ell$ and $p$ are the radial and azimuthal indices, respectively, that fully describes their transverse profile. The exponential term $\exp [i\ell \varphi ]$ is responsible for a phase singularity along the optical axis, which causes a ring-like intensity distribution, and the topological charge $\ell$ is associated to an amount of orbital angular momentum (OAM) $\ell \hslash$ per photon^29^. The parameters $R(z)$, $\omega (z)$ and $\zeta (z)$ are defined as,

6 $$R(z)=z[1+{(\frac{{z}_{R}}{z})}^{2}],\,\omega (z)={\omega }_{0}\sqrt{1+{(\frac{z}{{z}_{R}})}^{2}},\,\zeta (z)=\arctan (\frac{z}{{z}_{R}})\,{\rm{and}}\,{z}_{R}=\frac{\pi {\omega }_{0}^{2}}{\lambda }$$

where ${\omega }_{0}$ is the Gaussian beam waist at $z=0$ and ${z}_{R}$ is known as the Rayleigh range. Figure 2(a) shows the phase profile of the $L{G}_{1}^{2}$ mode that clearly illustrates its azimuthal variation. Figure 2(b) shows its intensity distribution constituted by two concentric rings of varying intensity, which is more obvious in the 2-dimensional transverse plot (along the dashed line) shown if Fig. 2(c).

To compute the total area of an $L{G}_{p}^{\ell }$ mode at a given distance $z$, we can use the generalised definition of “spot size”, which is taken as the maximum area to where the beam’s intensity still has a significant value. The spot size in this case will take the form^30^ (see Supplementary Materials),

7 $${A}_{p\ell }(z,p,\ell )=\pi \omega {(z)}^{2}(2p+|\ell |+1).$$

Notice that the area, or spot size, increases linearly with the modal indices $p$ and $|\ell |$ and quadratically with the propagation distance $z$. Further more, modes with the same modal index $M=2p+|\ell |+1$ are characterised by the same spot size.

In regards to the $H{G}_{nm}$ modes, these can be described mathematically in terms of the Hermite Polynomials ${H}_{n}(x)$ and ${H}_{m}(y)$ as,

8 $$\begin{array}{ll}H{G}_{nm}(x,y,z)= & \frac{1}{\omega (z)}\sqrt{\frac{{2}^{-(n+m-1)}}{\pi n!m!}}\exp [i(n+m+1)\zeta (z)]{H}_{n}[\frac{\sqrt{2}x}{\omega (z)}]\\  & {H}_{m}[\frac{\sqrt{2}y}{\omega (z)}]\,\exp \,[-\frac{{x}^{2}+{y}^{2}}{{\omega }^{2}(z)}]\,\exp \,[\frac{-ik({x}^{2}+{y}^{2})}{2R(z)}]\,\exp [-\,ikz],\end{array}$$

where, $R(z)$, ${z}_{R}$, $\omega (z)$, $\zeta (z)$ and ${\omega }_{0}$ are defined as in the previous case. Figure 2(d) shows the phase profile of the $H{G}_{23}$ mode, which also features an intricate pattern of non-constant phase. Its intensity distribution is shown in Fig. 2(e) where a non-homogeneous intensity pattern of bright and dark areas can be seen. This is better appreciated in the 2D plot shown in Fig. 2(f), taken along the dashed line shown in Fig. 2(e). In this case, the total area illuminated by a given $H{G}_{nm}$ mode can be computed as^31^ (see Supplementary Material),

9 $${A}_{nm}{(z,n,m)}_{n}=\omega {(z)}^{2}{[(2n+1)(2m+1)]}^{1/2}.$$

## Results

In this section we provide with experimental evidence that the non-homogeneous intensity distribution does not influence the speckle size. More precisely, we will show that the speckle produced by structured light modes has the same mean size as the one produced by plane waves of homogeneous intensity and constant phase. As a first example we compared a set of $L{G}_{p}^{\ell }$ modes generated by combinations of $\ell \in [0,5]$ and $p\in [0,5]$ with a subset of circular apertures of similar size. As a second example, we consider the set of $H{G}_{nm}$ modes generated by combinations of $n,m\in [0,5]$ and compared their speckle size with a subset of Rectangular apertures of similar size.

### Speckle size for Laguerre-Gaussian modes and circular apertures

**Figure 3:**
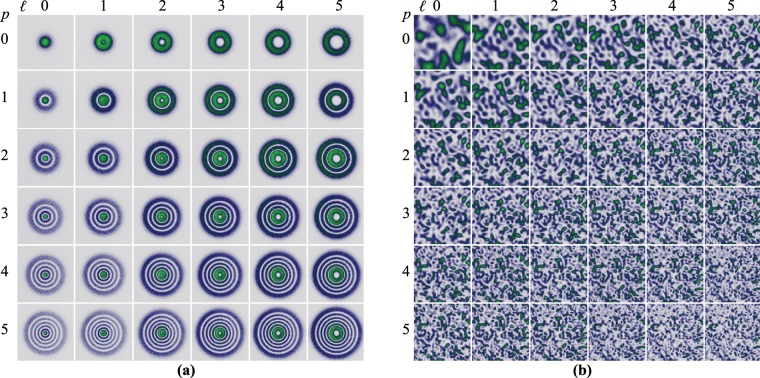
(**a**) Intensity profile of the subset of $L{G}_{p}^{\ell }$ modes given by combinations of $p\in [0,5]$ and $\ell \in [0,5]$. (**b**) Speckle intensity produced by the $L{G}_{p}^{\ell }$ shown in (**a**). Notice that the mean speckle size decreases as the spot size of the beam increases.

The intensity distribution of a subset of 36 $L{G}_{p}^{\ell }$ modes that were employed in the first experiment are shown in Fig. 3(a). This were generated using complex amplitude modulation encoded on a phase-only spatial light modulator^32,33^. The images were recorded with a Charge-Couple Device (CCD) camera (6.5 $\mu \,$m pixel size) at the plane $z=0$, precisely where the ground glass was positioned for the experiments. As can be seen, their spot size increases with $p$ and $\ell$, as predicted by Eq. 7. Figure 3(b) shows a section of the speckle obtained for each of the $L{G}_{p}^{\ell }$ modes shown in Fig. 3(a), which clearly evinces a decrease in size as the spot size of the $L{G}_{p}^{\ell }$ modes increases (See Methods for further details on the generation of speckle).

**Figure 4:**
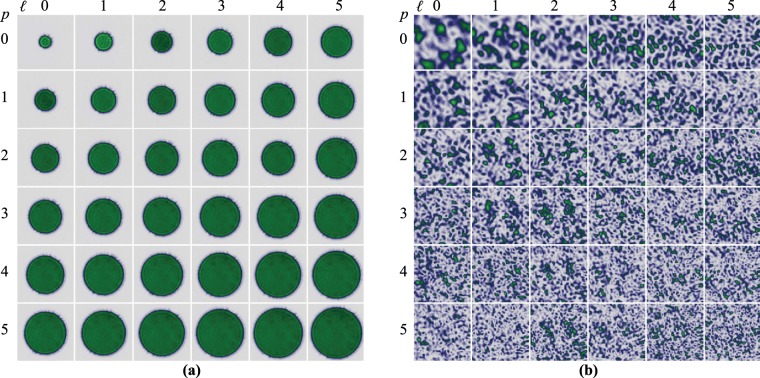
(**a**) Intensity profile of a set of 36 circular apertures of homogeneous intensity and radius $R={\omega }_{0}\sqrt{2p+|\ell |+1}$. (**b**) Speckle intensity produced by the intensity distribution of the apertures shown in (**a**). Notice how the speckle size decreases as the area of the circular aperture increases.

To compare our previous results, a set of 36 apertures of homogeneous intensity, also generated with the SLM, was used to illuminated the same region of the ground glass. The size of the circular apertures were chosen to match the spot size of the different $L{G}_{p}^{\ell }$ modes, for this, their radius were defined in terms of the modal number M as $R={\omega }_{0}\sqrt{2p+|\ell |+1}$, for example, the circular aperture defined by $p=1$ and $\ell =5$ illuminates the same area as the $L{G}_{1}^{5}$. Figure 4(a) shows the recorded intensities of the circular apertures at the plane $z=0$, to highlight that their radius are given in terms of the $L{G}_{p}^{\ell }$ modes they were labelled using the modal indices $p$ and $\ell$. Figure 4(b) shows, for comparison, the recorded speckle for this case, as expected, the speckle size decreases as the illuminated area increases.

Our previous result show that indeed, the mean speckle size produced by both, the $L{G}_{p}^{\ell }$ modes (Fig. 3) and the circular apertures (Fig. 4) decreases as the illuminated area increases. Nonetheless, a quantitative measure is required to show that the mean speckle size under both illumination sources is the same. To this end, we measured experimentally the mean size of the speckle produced by $L{G}_{p}^{\ell }$ modes using a numerical autocorrelation of each speckle image with itself (see Methods). To compare this with a theoretical model, we propose that the mean speckle size can be measured directly from Eq. 3, upon substitution of the area ${A}_{circ}$ by the spot size of the corresponding $L{G}_{p}^{\ell }$ modes, that is, (see Supplementary Materials),

10 $$\Delta {s}_{LG}(r)=\frac{1.22\lambda {z}_{f}}{2\omega (z){(2p+|\ell |+1)}^{1/2}}.$$

**Figure 5:**
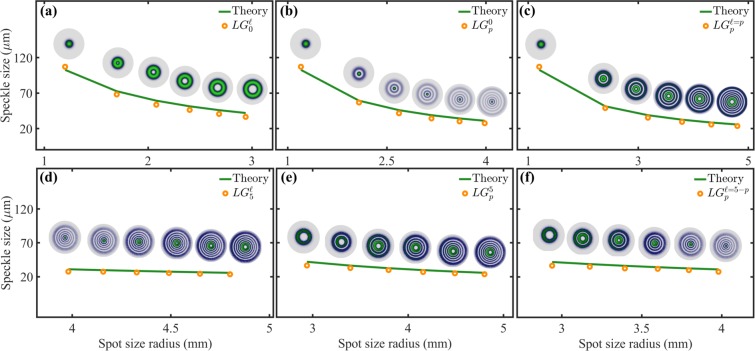
Speckle size as a function of the spot size for $L{G}_{p}^{\ell }$ modes, the theory (Eq. 10) is represented by green solid lines whereas the experiment by orange circles. In (**a**) and (**d**) $p=0$ and 5, respectively, for $\ell \in [0,5]$. In (**b**) and (**e**) $\ell =0$ and 5, respectively, for $p\in [0,5]$. In (**c**) $\ell =p$, while in (**f**) $\ell =5-p$ for $p\in [0,5]$.

As expected, this equation predicts that mean size of the speckle produced by $L{G}_{p}^{\ell }$ modes decreases as any of the two modal indices $\ell$ or $p$ increase. A comparison between our experimental measurements and our theoretical model (Eq. 10) is shown in Fig. 5. Here, theory is represented by green solid lines while the experiment by red points. First, in Fig. 5(a,d) we show the cases of constant radial indices, $p=0$ and $p=5$, respectively, and varying azimuthal indices $\ell \in [0,5]$, whereas in Fig. 5(b,e) we show the opposite case, that is $\ell =0$ and $\ell =5$, respectively, for $p\in [0,5]$. Finally, in Fig. 5(c,f) we show the most general cases where both, $\ell$ and $p$ increase. Notice the high correlation between our experimental measurements and the theoretical prediction, which confirms that indeed the inner structure of the $L{G}_{p}^{\ell }$ does not influence the mean speckle size.

**Figure 6:**
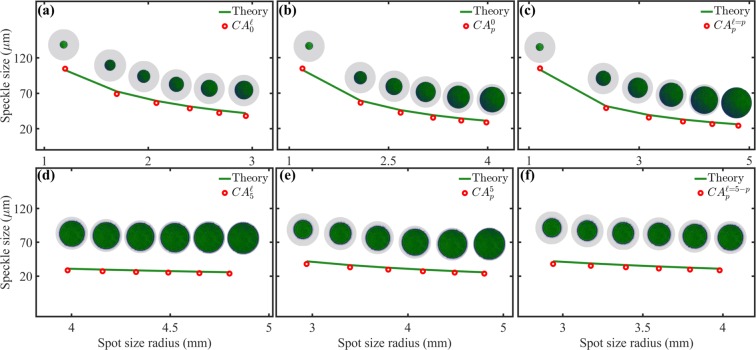
Speckle size as a function of the spot size for circular apertures of homogeneous intensity, the theory (Eq. 3) is represented by green solid lines whereas the experiment by red circles. The area of the circular apertures ($C{A}_{p}^{\ell }$) was chosen to match the spot size of the $L{G}_{p}^{\ell }$ modes. In (**a**) and (**d**) $p=0$ and 5, respectively, for $\ell \in [0,5]$. In (**b**) and (**e**) $\ell =0$ and 5, respectively, for $p\in [0,5]$. In (**c**) $\ell =p$, while in (**f**) $\ell =5-p$ for $p\in [0,5]$.

For the sake of completeness, we also computed the mean speckle size produced by the circular apertures of homogeneous intensity. Our results are shown in Fig. 6 where we plotted the mean speckle size as function of the radius of the area illuminated by the plane wave. Again, the theoretical prediction, given by Eq. 3, is plotted as a green solid line, whereas the experimental data by red points. The apertures of homogeneous intensity were labelled as $C{A}_{p}^{\ell }$ to emphasise again that their areas were chosen to match the spot size of the $L{G}_{p}^{\ell }$ modes. Further, the values of $p$ and $\ell$ where chosen exactly as in the $L{G}_{p}^{\ell }$ modes to make a fair comparison between both, $L{G}_{p}^{\ell }$ modes and circular apertures.

**Figure 7:**
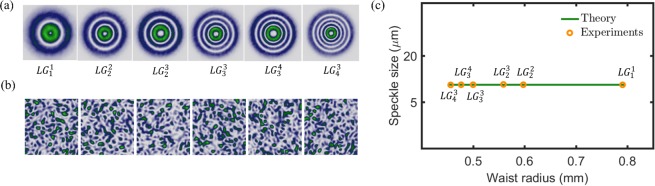
Speckle size for $L{G}_{p}^{\ell }$ modes of different beam waist but identical spot size. (**a**) Intensity distribution, (**b**) speckle intensity and (**c**) mean speckle size as function of the beam waist ${\omega }_{0}$.

We performed additional experiment to corroborate that indeed the mean speckle size only depends on the area illuminated by the $L{G}_{p}^{\ell }$ modes regardless of its internal structure. For this, we selected $L{G}_{p}^{\ell }$ modes of different modal numbers and modified their beams waist according to Eq. 7 to acquire the same spot size. Under this condition, even though they have different modal numbers, the spot size wil be the same for all. Figure 7(a) shows the intensity profile of the $L{G}_{p}^{\ell }$ modes used for this experiment, namely, $L{G}_{1}^{1}$, $L{G}_{2}^{2}$, $L{G}_{2}^{3}$, $L{G}_{3}^{3}$, $L{G}_{3}^{4}$, and $L{G}_{4}^{3}$. Accordingly, the corresponding beam waist for each mode is ${\omega }_{0}$, $2{\omega }_{0}/\sqrt{7}$, ${\omega }_{0}/\sqrt{2}$, $2{\omega }_{0}/\sqrt{11}$, ${\omega }_{0}/\sqrt{3}$, where ${\omega }_{0}=0.79$. Under this condition, all the beams should generate a speckle pattern with a similar mean size. Figure 7(b) shows the intensity distribution of the generated speckle for each case, which shows that the size is almost the same. This is more obvious in Fig. 7(c) where we plotted the mean speckle size as function of the beam waist for the different $L{G}_{p}^{\ell }$ modes. As can be seen, the mean speckle size ranges around 10.5 $\mu \,$m for all the modes. Additional results showing that $L{G}_{p}^{\ell }$ modes with the same spot size yield the same mean speckle size are shown in the Supplementary Materials.

### Speckle size for Hermite-Gaussian modes and rectangular apertures

**Figure 8:**
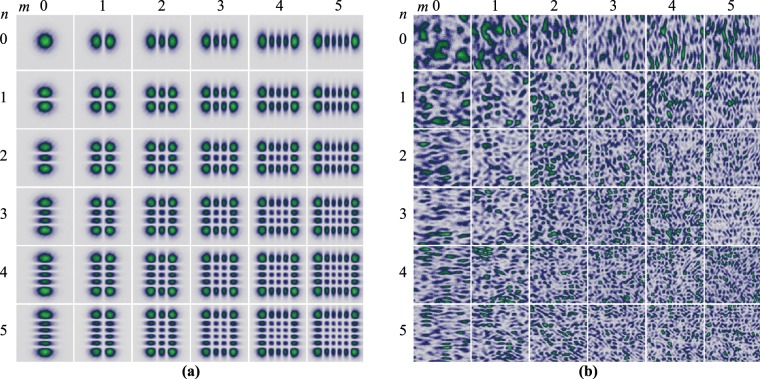
(**a**) Intensity distribution of the subset of $H{G}_{nm}$ modes given by combinations of $n,m\in [0,5]$. (**b**) Intensity of the speckle produced by the $H{G}_{nm}$ shown in (**a**). Notice that $H{G}_{nm}$ modes with rectangular shapes feature a elliptical shape, in the direction perpendicular the the light mode.

As a second example to reinforce our statement that the structure of light does not influence the mean speckle size, we considered the set of Hermite-Gaussian modes generated by combinations of $n,m\in [0,5]$, with their intensity profile shown in Fig. 8(a). As expected, an increase of the modal numbers produces an increase in the total area of the beam. The speckle generated by this subset of $H{G}_{nm}$ modes is shown in Fig. 8(b) where an decrease of the speckle size as function of the spot size can also be observed. Notice that modes with rectangular shape produce speckle with elliptical shape in the orthogonal direction, see for example the cases $H{G}_{05}$ and $H{G}_{50}$.

**Figure 9:**
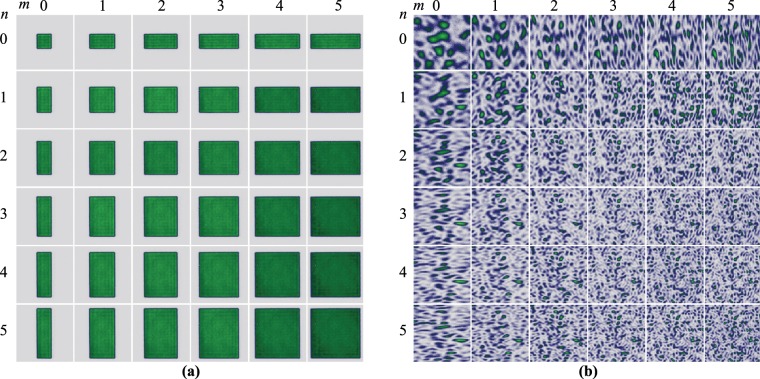
(**a**) Intensity distribution of a set of rectangular apertures of area ${A}_{nm}={\omega }_{0}^{2}{[(2n+1)(2m+1)]}^{1/2}$ and (**b**) the speckle produced by each of these.

Again, in order to compare the speckle generated by $H{G}_{nm}$ modes, we generated a set of homogeneous-intensity rectangular apertures of comparable dimensions. Figure 9(a) shows the intensity distribution of a set of rectangular apertures whose dimensions were defined in terms of the modal indices $m$ and $n$ as, ${L}_{x}={\omega }_{0}(2m+1)$ and ${L}_{y}={\omega }_{0}(2n+1)$. The speckle generated by these structures is shown in Fig. 9(b), as expected, the speckle size decreases as the area of the rectangular aperture increases. Further, in a similar way to the $H{G}_{nm}$ modes, the speckle generated by rectangular structures features also an elliptical shape in the perpendicular direction.

Again, to quantitatively compare the speckle size in both cases, we measured the speckle size and plotted this as function of the spot size of $H{G}_{nm}$. In this case, the mean speckle size as function of the area illuminated by the $H{G}_{nm}$ can be computed from the relation (see Supplementary Materials),

11 $$\Delta {s}_{HG}=\frac{\lambda {z}_{f}}{2\omega (z){[(2n+1)(2m+1)]}^{1/4}},$$

which is in essence Eq. 4, where the area illuminating the ground glass has been taken as the spot size of the $H{G}_{nm}$ modes (Eq. 9).

**Figure 10:**
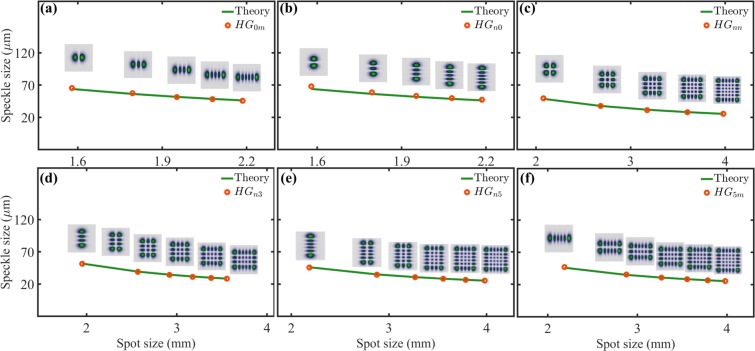
Speckle size as function of the illuminated area for $H{G}_{nm}$ modes. The theory is represented by green solid lines ant the experimental data by orange circles. In (**a**) $n=0$ for $m\in [1,5]$. In (**b**) and (**c**) $m=0$ and $m=n$, respectively, for $n\in [1,5]$. In (**d**) and (**e**) $m=3$ and $m=5$, respectively, for $n\in [0,5]$. In (**f**) $n=5$ for $m\in [0,5]$.

**Figure 11:**
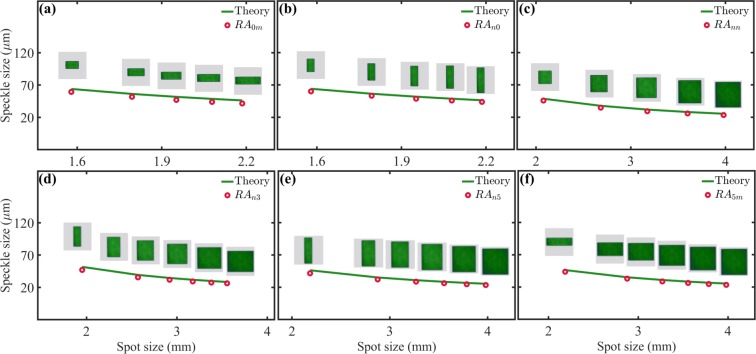
Speckle size as function of the illuminated area for plane waves of rectangular shape and similar dimensions to the $H{G}_{nm}$ modes. In (**a**) $n=0$ for $m\in [1,5]$. In (**b**,**c**) $m=0$ and $m=n$, respectively, for $n\in [1,5]$. In (**d**,**e**) $m=3$ and $m=5$, respectively, for $n\in [0,5]$. In (**f**) $n=5$ for $m\in [0,5]$.

Figure 10 shows a comparison of both, theory (green solid line) and experimental measurements (orange circles). The case $n=m=0$ is the fundamental Gaussian beam with circular symmetry and was not included here. In Fig. 10(a,f) we show the cases $H{G}_{0m}$ and $H{G}_{5m}$, respectively, for $m\in [1,5]$ whereas in Fig. 10(b,d,e), show the cases $H{G}_{n0}$, $H{G}_{n3}$ and $H{G}_{n5}$, respectively. Finally Fig. 10(c) shows the case $H{G}_{nm}$, where, $n=m$ and, $m\in [1,5]$. Notice that in all cases the speckle size predicted by Eq. 11, matches almost perfectly the speckle size measured experimentally. As with the case of $L{G}_{p}^{\ell }$ modes, we also measured the mean size of the speckle produced by the rectangular apertures. Our results are shown in Fig. 11, the mean speckle size plotted as function of the radius of the illuminated area. The theory, as predicted by Eq. 4, is plotted as a green solid line, whereas the experiments by red circles. Notice again that, as expected, the experiments matched almost exactly the theoretical predictions. This is not surprising since Eq. 4 was precisely derived for rectangular apertures of homogeneous intensity.

## Discussion

In this manuscript we demonstrated that the mean size of the speckle produced by structured light modes of non-homogeneous intensity and phase distributions, is not influenced by their complex structure and therefore, its mean size can be related to the total illuminated area. A possible explanation for this lies in the fact that at the scattering surface, each scatter acts as a point source, where light is absorbed and re-emitted with a random phase. In other words, the coherent light after striking or passing through the scattering surface losses its coherence. Since the speckle is generated from the interference of a large number of these scatters, the original structure of the light mode becomes irrelevant. To corroborate this, we derived simple mathematical expressions that relate the mean speckle size to the the total area illuminated by the structured light mode, known as spot size. To prove this assertion, we exploited the complex structure of the Laguerre- and the Hermite-Gaussian modes, characterised by non-homogeneous intensity and phase distributions. We generated these modes experimentally and analysed the speckle produced by a ground glass plate. We also generated circular and rectangular apertures of homogeneous intensity distribution to compare the size of the generated speckle with the structured light modes. First, we measured experimentally the speckle size produced by a subset of $L{G}_{p}^{\ell }$ modes, using image correlation, and compared this with the speckle produced by circular apertures of similar size, finding a perfect mach between both cases. This finding allowed us to provide with a mathematical expression that relates the mean speckle size with the total area illuminated by a light mode, computed through the generalised definition of spot size. Crucially, the speckle size measured experimentally, fits very well to our proposed mathematical expression, evincing that indeed, the structure of light does not affect the statistical properties of speckle. To further support this evidence, we also measured the speckle size produced by a subset of $H{G}_{nm}$ and compared this with the one produced by rectangular apertures of homogeneous intensity, finding again a perfect match between both cases. Our proposed mathematical expressions to measure the speckle size of $H{G}_{nm}$ modes also fits very well to the experimental results. Our findings are of paramount importance, since they reveal a property of speckle that, to the best of our knowledge, has never been reported. Further, they will pave the way towards the development of novel applications. For example, in optical metrology, where it has been already shown that the roughness of a surface can be measured with higher sensitivities using the speckle produced by optical vortices^34^. In addition, nondiffracting speckle, produced also with optical vortices, shows great potential in the testing of materials as well as in optical tweezers^17^. In addition, the generation of oriented speckle, which can be produced and controlled with Hermite-Gaussian modes, has applications in data storage^26^.

## Methods

**Figure 12:**
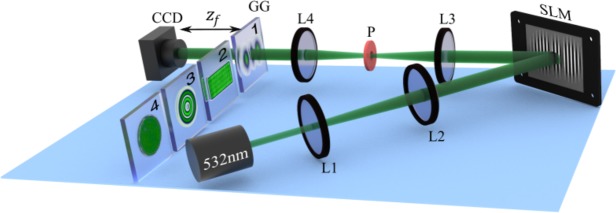
Schematic representation of the experimental setup implemented to measure the mean speckle size. SLM: Spatial Light Modulator, L1-L4: Lens, P: Pinhole, CCD: Charge-Coupled Device camera. The insets labelled as 1, 2, 3 and 4 show examples of the beam’s intensity profile that illuminated the ground glass (GG).

In order to show that indeed the structure of light does not influence the speckle size, we performed a series of experiments using the experimental setup depicted in Fig. 12. A continuous wave (CW) laser ($\lambda =532$ nm) was expanded, using lenses L1 ($f=25$ mm) and L2 ($f=150$ mm), to approximate a flat wave front and redirected onto a Spatial Light Modulator (SLM, Holoeye Pluto $1920\times 1080$, $8\mu \,$m pixel size). The $L{G}_{p}^{\ell }$ and $H{G}_{nm}$ modes shown in Figs. 3(a) and 8(a), respectively, as described by Eqs. 5 and 8, where we used ${\omega }_{0}=300\,\mu {\rm{m}}$. In addition, we also encoded on the SLM the circular and rectangular apertures shown in Figs. 3(b) and 8(b), respectively. Once generated, the modes were expanded two times and imaged onto a ground glass plate (AT1 from Thorlabs), using a 4 f imaging system composed of lenses L3 ($f=100$ mm) and L4 ($f=200$ mm). The purpose of the image system is to relay the plane of the SLM to the plane of the ground glass (GG). A spatial aperture (P) placed between L3 and L4 enabled the removal of higher diffraction orders. The insets labelled as 1, 2, 3 and 4, show some examples of the beams illuminating the ground glass. The speckle was then measured at a distance ${z}_{f}$ from the ground glass and recorded with the CCD.

**Figure 13:**
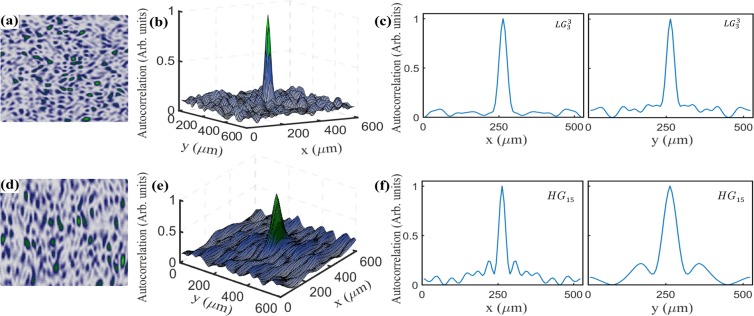
(**a,d**) Show two examples of the speckle generated by the modes $L{G}_{3}^{3}$ and $H{G}_{15}$, respectively. (**b**,**e**) Numerical autocorrelation of the images shown in (**a**,**d**), respectively. (**c**,**f**) Show the 2D profile along the $x$ and $y$ directions. Notice the vertical elliptical shape of the $H{G}_{15}$ mode and of its numerical autocorrelation.

To measure the mean speckle size, we performed a numerical autocorrelation of each speckle image with itself. Figure 13(a) shows an example of the speckle generated with and $L{G}_{3}^{3}$ mode, while Fig. 13(b) shows a 3D image of the numerical autocorrelation, clearly showing a central peak corresponding to the complete overlapping of speckle. The 2D plots shown in Fig. 13(c) display the transverse profile along the $x$ and $y$ directions, respectively. Figure 13(d) shows an example of the speckle generated by the $H{G}_{15}$ modes, which features an elliptical shape along the vertical direction. Of course, the shape of the speckle produces also an autocorelation with an elliptical shape, as shown in 13(e) and the 2D plot of Fig. 13(f). From the plots shown in 13(c,f), we measured the speckle size as the full width at half-maximum (FWHM).

## Supplementary information

Supplementary Information.

## Data Availability

All data regarding the work presented here is available upon reasonable request to the corresponding author.
