# Supplementary material for: Does the structure of light influence the speckle size?
Source: Sci Rep. 2020 Jan 13;10:199. doi: 10.1038/s41598-019-56964-0 (PMC6957527; doi:10.1038/s41598-019-56964-0)
Supplement: Supplementary file 1 — Supplementary Information. [file 41598_2019_56964_MOESM1_ESM.pdf]

Does the structure of light influence the speckle size? Supplementary material

Xiao-Bo Hu[1,2], Meng-Xuan Dong[2], Zhi-Han Zhu[2], Wei Gao[2], and Carmelo Rosales-Guzmán[2,*]

[1]The Higher Educational Key Laboratory for Measuring & Control Technology and Instrumentations of Heilongjiang Province, Harbin University of Science & Technology, Harbin 150080, China
[2]Wang Da-Heng Collaborative Innovation Center, Heilongjiang Provincial Key Laboratory of Quantum manipulation & Control, Harbin University of Science and Technology, Harbin 150080, China
[*]Corresponding author:carmelorosalesg@hrbust.edu.cn

ABSTRACT

Here we provide with additional information about the implementation of the autocorrelation function used in our main text to measure the speckle size of circular and rectangular apertures of homogeneous intensity. We also provide with a brief description about the generalised definition of spot size, which allows to measure the total area illuminated by the LG_p^ℓ and HG_{nm} modes. Finally, we provide with more examples of the speckle generated by LG_p^ℓ modes of identical spot size to highlight the fact that regardless of the difference in their phase and intensity distribution, the mean size of the generated speckle is almost identical.

Speckle size produced by circular and rectangular aperture of homogeneous-intensity

The mean speckle size can be measured through the autocorrelation function, which in terms of the intensity of the beam illuminating the rough surface is given by[1],

$$\mathrm{C}(\Delta u,\Delta v)=\langle I\rangle^2\left[1+\left|\frac{\iint_{-\infty}^{+\infty}|\mathrm{P}(x,y)|^2\exp\left[\frac{i2\pi}{\lambda z_f}(x\Delta u+y\Delta v)\right]\mathrm{d}x\mathrm{d}y}{\iint_{-\infty}^{+\infty}|\mathrm{P}(x,y)|^2\mathrm{d}x\mathrm{d}y}\right|^2\right], \tag{1}$$

where, $|\mathrm{P}(x,y)|^2$ is the intensity function describing the area illuminated by the light beam. For the specific case of a circular aperture of radius R and uniform intensity given by,

$$|\mathrm{P}_{circ}(x,y)|^2=\mathrm{circ}\left(\frac{\rho}{R}\right), \tag{2}$$

where $\rho=\sqrt{x^2+y^2}$ and circ(ρ/R)=1 for $|\rho|\leq R$ and zero otherwise, we obtain, after substituting into Eq. 1, the well-known expression[2],

$$\mathrm{C}_{circ}(\Delta u,\Delta v)=\langle I\rangle^2\left[1+\frac{r^3}{\lambda z_f}\left|\frac{\mathrm{J}_1\left(\frac{2\pi r}{\lambda z_f}\sqrt{\Delta u^2+\Delta v^2}\right)}{\sqrt{\Delta u^2+\Delta v^2}}\right|^2\right]. \tag{3}$$

In the above equation, z_f is the distance between the rough surface and the observation plane and $\mathrm{J}_1(\xi)$ is the Bessel function of the first kind and order 1. The mean radius of the speckle can then be definedn as the value $\sqrt{\Delta u^2+\Delta v^2}\equiv\Delta s_{circ}$ for which $\mathrm{J}_1(\xi)$ first becomes zero, which happens when the argument is equal to 1.22π. Hence, the mean diameter of the generated speckle is,

$$\Delta s_{circ}=\frac{1.22\lambda z_f}{2R} \tag{4}$$

The above equation can be written in terms of the total illuminated area as,

$$\Delta s_{circ}=\frac{1.22\sqrt{\pi}\lambda z_f}{2\sqrt{A}}, \tag{5}$$

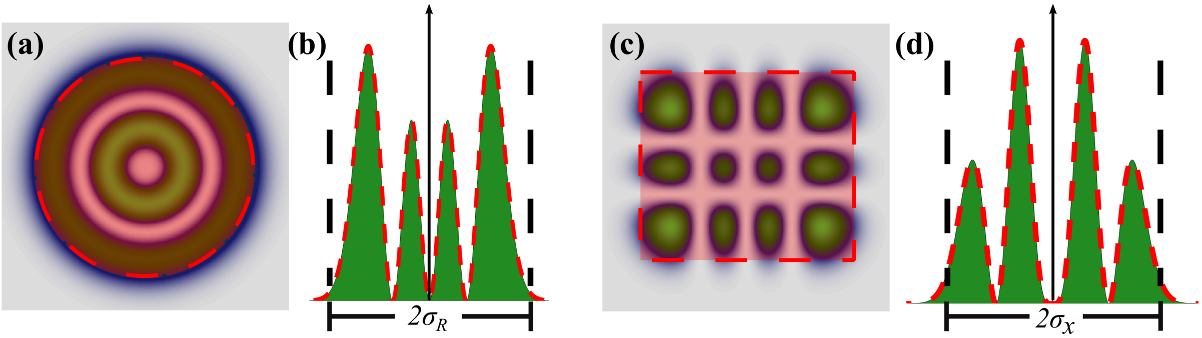

Figure S1. Intensity distribution of an LG_1^2 mode, whose spot size is illustrated by the transparent circle in (a), which is also indicated by the dashed vertical lines shown in the 1-Dimensional intensity profile in (b). Intensity distribution of an HG_{23} mode, whose spot size is illustrated by the transparent rectangle (c), also illustrated in the 1-Dimensional intensity profile shown in (d).

which is Eq.2 of our main manuscript.

For the case when the illuminating wave has a rectangular shape of dimensions $L_1 \times L_2$, the intensity function is mathematically given by,

$$\mathrm{P}_{rect}(x,y)|^2 = \mathrm{rect}\left(\frac{x}{L_x}\right)\mathrm{rect}\left(\frac{y}{L_y}\right), \tag{6}$$

where, rect(x)=1 for $|x| \leq 1$ and zero otherwise. In this case, the autocorrelation function, obtained by inserting Eq. 6 into Eq. 1 will take the form[1,2],

$$\mathrm{C}_{rect}(\Delta u, \Delta v) = \langle I \rangle^2 \left[1 + \mathrm{sinc}^2\left(\frac{L_x \Delta u}{\lambda z_f}\right)\mathrm{sinc}^2\left(\frac{L_y \Delta v}{\lambda z_f}\right)\right], \tag{7}$$

where sinc(ξ) is defined as $\sin(\pi\xi)/(\pi\xi)$. Again, the mean speckle size can then be taken as the value Δu (or Δv) for which $\mathrm{sinc}^2(\xi)$ first become zero, which yields,

$$\Delta s_x = \frac{\lambda z_f}{L_x} \quad \text{and} \quad \Delta s_y = \frac{\lambda z_f}{L_y} \tag{8}$$

In terms of the total illuminated area $A = L_x L_y$, the mean speckle size is then defined as

$$\Delta s_{rect} = \frac{\lambda z_f}{\sqrt{L_x L_y}}, \tag{9}$$

which is Eq. 4 in our main text.

The spot size in Laguerre- and Hermite-Gaussian modes

The generalised definition of "spot size", taken as the maximum area to where the beam's intensity still has a significant value, provides with the perfect means to measure the total area illuminated by the LG_p^ℓ and HG_{nm} modes (see Fig. S1). For the case of LG_p^ℓ modes, the radius σ of the spot size (see Fig. S1(a) and S1(b)) can be derived using the standard deviation as[3],

$$\sigma_{p\ell}^2(z,p,\ell) = \frac{2\int_0^{2\pi}\int_0^\infty r^2 I_{LG}(x,y,z)\mathrm{d}\rho\mathrm{d}\theta}{\int_0^{2\pi}\int_0^\infty I_{LG}(x,y,z)\mathrm{d}\rho\mathrm{d}\theta}, \tag{10}$$

where, $I_{LG}(x,y,z)$ is the intensity of a given LG_p^ℓ mode. Both integrals in Eq. 10 are straightforward and the final result provides with the spot size radius in terms of both modal indices (see[3] for a detailed derivation), namely,

$$\sigma_{p\ell}(z,p,\ell) = \omega(z)(2p + |\ell| + 1)^{1/2}. \tag{11}$$

Hence, the spot size for any LG_p^ℓ mode, as function of the modal indices takes the form,

$$A_{p\ell}(z,p,\ell) = \pi\omega(z)^2(2p + |\ell| + 1), \tag{12}$$

(μm)	$l=0$	$l=1$	$l=2$	$l=3$	$l=4$	$l=5$
$p=0$	104.77	69.07	56.37	48.66	42.30	37.98
$p=1$	56.36	48.67	42.27	37.96	35.23	33.12
$p=2$	42.29	37.95	35.22	33.12	31.21	29.70
$p=3$	35.25	33.12	31.21	29.70	28.57	27.23
$p=4$	31.22	29.70	28.56	27.22	26.10	25.28
$p=5$	28.58	27.23	26.10	25.27	24.43	23.78

Figure S2. Mean speckle size generated by the subset of LG_p^ℓ modes analysed in our main text. Here modes with the same spot size have been coded with the same colour. Notice that the mean speckle size labelled with the same colour is almost identical.

which is equation 7 of our main file.

For the case of HG_{nm} modes (S1(c) and S1(d)), the spot size can be found by first computing the standard deviation along the horizontal and vertical directions[4], that is,

$$\sigma_x^2(z,n) = \frac{2\int\int_{-\infty}^{\infty} x^2 I_{n0}(x,y,z)\mathrm{d}xdy}{\int\int_{-\infty}^{\infty} I_{n0}(x,y,z)dxdy}, \qquad \sigma_y^2(z,m) = \frac{2\int\int_{-\infty}^{\infty} y^2 I_{0m}(x,y,z)dxdy}{\int\int_{-\infty}^{\infty} I_{0m}(x,y,z)dxdy}. \tag{13}$$

where, $I_{0m}(x,y,z)$ and $I_{n0}(x,y,z)$ are the intensities of the $HG_{0m}(x,y,z)$ and $HG_{n0}(x,y,z)$ modes, respectively. The integrals in Eq. 13 can be computed in a straight forwad way, yielding the final result[3],

$$\sigma_x(z)_n = \omega(z)(2n+1)^{1/2}, \qquad \sigma_y(z)_m = \omega(z)(2m+1)^{1/2}, \tag{14}$$

from which, the spot size can be computed as,

$$A_{nm}(z,n,m) = \omega(z)^2[(2n+1)(2m+1)]^{1/2}, \tag{15}$$

which is Eq. 9 in our main file.

Speckle size for Laguerre-Gaussian modes of similar areas

A closer inspection of Eq. 12 reveals that the spot size of certain LG_p^ℓ modes will be the same for particular combinations of p and ℓ. For example, the mode LG_0^2 has the same area as the mode LG_1^0, namely, $A_{p\ell} = 3\pi\omega(z)^2$. In the same way the modes LG_4^0, LG_3^2 and LG_2^4 will also have the same area, which in this case will take the value $A_{p\ell} = 9\pi\omega(z)^2$. Incidentally, modes with the same spot size will propagate in an identical manner, experiencing the same diffraction properties. It is expected then that such modes should produce a speckle pattern with the same mean size. This was corroborated experimentally and shown in Fig. S2 where a detailed list of the mean speckle size for the subset of LG_p^ℓ analysed in the main text is presented. For the sake of clarity, modes with the same spot size are represented with the same colour. For example, the modes LG_3^0, LG_2^2 and LG_1^4, with the same spot size, have almost identical mean speckle size, namely, 35.25 μm, 35.22μm and 35.23μm, respectively.

References

1. Goodman, J. W. *Laser Speckle and Related Phenomena*, vol. 9 (Springer-Verlag Berlin Heidelberg, 1975).
2. Voelz, D. G. *Computational Fourier Optics: A MATLAB Tutorial*, vol. TT89 of *Tutorial Texts (Book 89)* (SPIE Press, 2011).
3. Phillips, R. L. & Andrews, L. C. Spot size and divergence for laguerre gaussian beams of any order. *Appl. Opt.* **22**, 643–644, DOI: 10.1364/AO.22.000643 (1983).
4. Carter, W. H. Spot size and divergence for hermite gaussian beams of any order. *Appl. Opt.* **19**, 1027–1029, DOI: 10.1364/AO.19.001027 (1980).
